# Association Between Environmental and Socioeconomic Risk Factors and Hepatocellular Carcinoma: A Meta-Analysis

**DOI:** 10.3389/fpubh.2022.741490

**Published:** 2022-02-18

**Authors:** Wenfeng Lu, Fengjiao Zheng, Zhi Li, Rui Zhou, Lugang Deng, Wenwei Xiao, Wenyan Chen, Rong Zhao, Yulan Chen, Yuxing Tan, Zhibo Li, Limin Liu, Duxun Tan, Nan Liu

**Affiliations:** ^1^Institute of Environment and Health, Health Science Center, South China Hospital, Shenzhen University, Shenzhen, China; ^2^College of Public Health, Zhengzhou University, Zhengzhou, China; ^3^Department of Clinical Laboratory, The Air Force Hospital of Southern Theater Command of Chinese People's Liberation Army (PLA), Guangzhou, China; ^4^Institute of Chronic Disease Risks Assessment, School of Nursing and Health, Henan University, Kaifeng, China

**Keywords:** environmental factor, hepatocellular carcinoma, liver cancer, meta-analysis, risk factors

## Abstract

**Background:**

The association between environmental and socioeconomic risk factors and the occurrence of hepatocellular carcinoma (HCC) are still inconclusive. A meta-analysis was conducted to address this issue.

**Methods:**

We systematically searched the databases including PubMed, Web of Science, and Google Scholar and collected the related risk factors of HCC before March 6, 2020. Statistical analysis was performed on the odds ratio (OR) value and 95% CI of the correlation between environmental and socioeconomic factors and HCC. Begg's rank correlation test, Egger's linear regression test, and the funnel plot were employed for identification of the publication bias.

**Results:**

Out of 42 studies, a total of 57,892 participants were included. Environmental and socioeconomic risk factors including ever educated (illiteracy); race (Black, Hispanic, and Asian); medium and low incomes; occupations (farmer and labor); passive smoking; place of residence (rural); blood aflatoxin B1 (AFB1) adduct level; exposure of pesticide, etc., were statistically increased with the occurrence of HCC (*P* < 0.05) and OR values and 95% CIs were 1.37 (1.00, 1.89), 2.42 (1.10–5.31), 1.90 (0.87–4.17), 5.36 (0.72–40.14), 1.48 (1.11, 1.96), 1.74 (1.00–3.03), 1.49 (1.06–2.08), 1.52 (1.07–2.18), 1.43 (0.27, 7.51), 1.46 (1.09, 1.96), 2.58 (1.67–3.97), and 1.52 (0.95–2.42), respectively. We found 6–9, 9–12, and ≥12 years of education that statistically reduced the risk of the occurrence of HCC (*P* < 0.05) and OR values and 95% CIs were 0.70 (0.58, 0.86), 0.52 (0.40, 0.68), and 0.37 (0.23, 0.59), respectively. No significant associations (*P* > 0.05) were observed between race (Hispanic and Asian), passive smoking, marital status, place of birth, place of residence, and HCC. In stratified analysis, exposure of pesticide was statistically significant (*P* < 0.05), while race of black was on the contrary.

**Conclusion:**

Environmental and socioeconomic risk factors have great impacts on the incidence rate of HCC. Improving national education and income levels can significantly reduce the risk of HCC.

**PROSPERO Registration:**

https://www.crd.york.ac.uk/prospero/, identifier: CRD42020151710.

## Introduction

Hepatocellular carcinoma (HCC) has become a growing global concern in the recent years. According to the Global Cancer Statistics 2020 by the International Agency for Research on Cancer, there are approximately 905,677 new cases of liver cancer and 830,180 deaths worldwide (1). The incidence rate of liver cancer ranks seventh among all the cancers in the world and mortality rate ranks second in men. HCC is a common cancer in many regions and countries including the United States (2), South Korea (3), and especially China (1). According to the latest National Cancer Statistics released by the Chinese National Cancer Center (4), about 364,800 new cases of liver cancer occurred in China in 2014, with the highest morbidity and mortality in less developed areas such as Western China. The younger tendency of HCC is more and more obvious, particularly in the population aged under 40-year-old. The 5-year survival rate of early liver cancer with surgical treatment is about 15%, while in the middle and advanced stages, it is even lower, which poses a huge threat to the physical and mental health of the human body (5).

The prevention of HCC is imminent and it is necessary to control the etiology that affects the incidence, especially the risk factors of liver cancer. There are three main categories of factors (6) affecting the occurrence of HCC including: environments (7), diets, and genetic factors. Passive smoking, aflatoxin B1 (AFB1) exposure, pesticide exposure, place of residence, schistosomiasis infection, etc., are considered as natural environmental factors and the social environmental factors are education, race, occupation, income, etc. The environment around us which we are relying to survive is the necessary prerequisite for the existence and development of human society. As a carrier of the daily life for the population, various factors in the environment affect our lives. By controlling the effects of environmental factors on the human body to reduce the incidence of HCC, it is one of the means in tertiary prevention. The aim of this study is to identify the association between environmental factors and HCC and clarify the factors that affect the incidence of liver cancer with updated literature. With the supporting factors, we now have a targeted approach to provide effective medical measurements in addressing this health program. Therefore, we can reduce the harm of HCC to the population, save medical resources, improve the physical health of people, and improve the quality of life of people.

## Methods

### Study Selection Criteria

Inclusion criteria: All the case–control studies associated with environmental factors and HCC were included in this study. Studies should report odds ratios (ORs) with corresponding 95% CIs or provide enough data to calculate. Exclusion criteria: (1) control population with additional liver diseases; (2) publications such as review, editorial, commentary, qualitative studies, and so on; (3) studies in language other than English; and (4) studies by using the same data. The protocol for this analysis was registered with the PROSPERO (CRD42020151710).

### Search Strategy

The meta-analysis was carried out according to the criteria of Preferred Reporting Items for Systematic Reviews and Meta-Analyses (PRISMA) (8). An electronic search for the case–control studies from inception until March 4, 2020 was performed among databases including PubMed, Web of Science, and Google Scholar, by using both the Medical subject headings (MeSH) terms and free terms such as education, race, ethnic, farmer, labor, second-hand smoke, passive smoking, place of birth, place of residence, marital status, married, rural region, urban region, pesticide, schistosome, occupation, income, AF, AFB1, environmental factor, socioeconomic factor, etc. The reference lists of relevant studies were screened additionally. All the studies were imported into document management software (Endnote, version X9, Thomson Scientific, Stamford, Connecticut, USA) for processing.

### Data Extraction

Study retrieval and data extraction were carried out in document management software by two researchers independently. Title, abstract, and full text of the studies were reviewed by using the selection criteria. A third researcher committed to resolve the difference through discussion. After screening, the data were extracted to evaluate the quality of the included studies and conduct for data analysis. The extracted information includes the following terms: author, publication year, research year, country, study design, number of the case and control groups, source of the control group, relative factors, OR, 95% CI, etc.

### Quality Evaluation

Study quality was evaluated mainly through the Newcastle–Ottawa scale (NOS) (9) to reduce the bias. It mainly confirms whether there are designing flaws or missing information between individual studies. The evaluator is required to take the NOS as the core and conduct a comprehensive evaluation (out of 9 points) at each grading point. Documents were excluded with lower scores for improving the credibility of this meta-analysis. Two researchers independently completed the evaluation and then conducted cross-comparison. Any dispute should be settled by a third investigator through consultation. Collected documents were divided into three categories: (1) a total score with ≥8 points can be defined as high-quality documents; (2) ≥6 points can be defined as general quality documents; and (3) ≤ 5 points can be defined as low-quality documents for exclusion, respectively (10).

### Statistical Analysis

In this study, the corresponding outcome variables and sum effect measures were employed for evaluating the different data extracted from the included studies. We took ORs for synthesis as measurement for the sum effect. Stata (version 15.0, StataCorp, College Station, Texas, USA) was used to analyze the outcome of OR, 95% CI, and the two-sided *P*-value for each result. Heterogeneity test for the included studies was checked by the *I*^2^ test with values of 25, 50, and 75% representing low, moderate, and high degrees, respectively (11). When *I*^2^ ≥ 50%, there was substantially heterogeneity in the study and a random-effects model was chosen. Otherwise, we would choose a fixed-effects model. We conducted a sensitivity analysis to find out the possible sources of heterogeneity. In addition, subgroup analysis was employed to explore the possible sources of heterogeneity based on sample size, study design, point of quality evaluation, the source of control populations, etc. To assess the publication bias, we obtained the continuous and binary outcomes by Egger's linear regression test and Begg's rank correlation test, respectively. More intuitive outcomes were also assessed by the funnel plot.

## Results

### Study Characteristics

**Figure 1:**
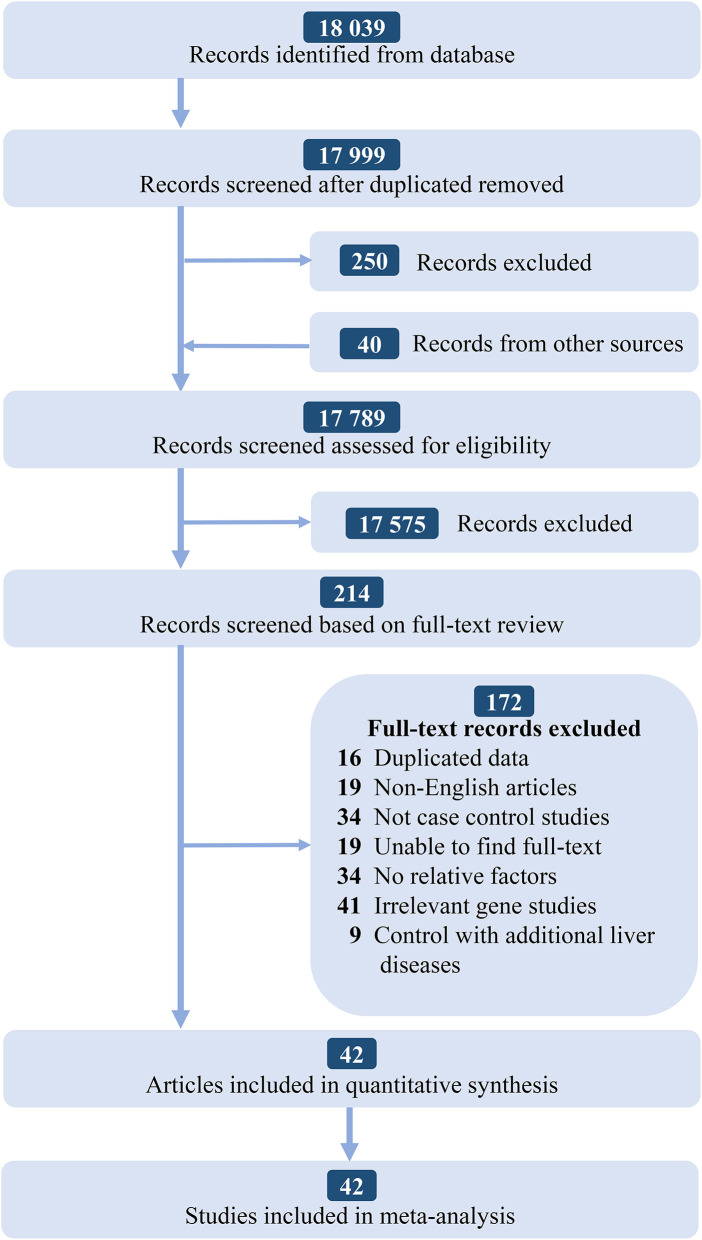
Flowchart of the literature selection searching process and selection strategy.

According to the PRISMA statement, there are four steps in total and the inclusion studies are strictly handled. The selection process and the results of the literature search are given in Figure 1. We identified 18,039 records from the initial screening. Finally, we found 42 studies that satisfying the selection criteria were included for the quantitative synthesis. The basic characteristics of included studies are shown in Supplementary Table 1.

### Quality Evaluation of Included Studies

The quality of the studies was evaluated through the NOS scale and the articles with a score >5 were included. The NOS scale was divided into three parts including selection, comparability, and exposure. Finally, 26 articles were defined as high-quality documents scored ≥8 and 12 articles scored ≥6. Additionally, 4 articles scored ≤ 5 were excluded (12–15). The details are given in Supplementary Table 1.

### Data Synthesis

In this meta-analysis, a total of 42 case–control studies with 57,892 participants were included. Table 1 demonstrates the heterogeneity test, which suggests that education (6–9 years: OR = 0.70, 95% CI: 0.58–0.86; 9–12 years: OR = 0.52, 95% CI: 0.40, 0.68; ≥12 years: OR = 0.37, 95% CI: 0.23–0.59); illiteracy (OR = 1.37, 95% CI: 1.00–1.89); race (Black: OR 2.42, 95% CI: 1.10–5.31, Hispanic: OR 1.90, 95% CI: 0.87–4.17, Asian: OR 5.36, 95% CI: 0.72–40.14); income (medium: OR 1.48, 95% CI: 1.11–1.96; low: OR 1.74, 95% CI: 1.00–3.03); occupation (farmer: OR 1.49, 95% CI: 1.06–2.08; labor: OR 1.52, 95% CI: 1.07–2.18); passive smoking (OR 1.43, 95% CI: 0.27, 7.51); marital status (married: OR 0.68, 95% CI: 0.36, 1.29); place of residence (rural: OR 1.46, 95% CI: 1.09, 1.96); blood AFB1 adduct level (OR 2.58, 95% CI: 1.67–3.97); and exposure of pesticide (OR 1.52, 95% CI: 0.95–2.42) are statistically significant (*P* < 0.05).

**Table 1:** The result of heterogeneity test.

**Factor**		**No. of studies**	**Heterogeneity test**		**Model**	**OR and 95%CI**
			***I^**2**^* (%)**	***P***		
Years of education	0, 0–6	17	–	–	–	1
	6–9	12	55.4	0.010	Random	0.70 (0.58, 0.86)^*^
	9–12	9	58.4	0.014	Random	0.52 (0.40, 0.68)^*^
	>12	7	56.1	0.033	Random	0.37 (0.23, 0.59)^*^
Ever educated (illiteracy)		11	85.6	0.000	Random	1.37 (1.00, 1.89)^*^
Race	White	5	–	–	–	1
	Black	5	95.2	0.000	Random	2.42 (1.10, 5.31)^*^
	Hispanic	3	93.5	0.000	Random	1.90 (0.87, 4.17)^*^
	Asian	3	98.7	0.000	Random	5.36 (0.72, 40.14)^*^
Income	High	9	–	–	–	1
	Medium	9	87.4	0.000	Random	1.48 (1.11, 1.96)^*^
	Low	9	95.8	0.000	Random	1.74 (1.00, 3.03)^*^
Occupation	farmer	13	80.6	0.000	Random	1.49 (1.06, 2.08)^*^
	Labor	6	71.3	0.004	Random	1.52 (1.07, 2.18)^*^
Passive smoking		3	96.8	0.000	Random	1.43 (0.27, 7.51)^*^
Marital status (married)		6	93.5	0.000	Random	0.68 (0.36, 1.29)^*^
Place of residence (rural)		5	61.3	0.035	Random	1.05 (0.76, 1.44)^*^
Place of birth (rural)		2	0.0	0.716	Fixed	1.46 (1.09, 1.96)
Blood AFB1 adduct level		6	77.6	0.000	Random	2.58 (1.67, 3.97)^*^
Urinary AFB1 albumin level		4	14.7	0.319	Fixed	2.16 (1.56, 3.00)
Exposure of pesticide		5	81.1	0.000	Random	1.52 (0.95, 2.42)^*^
Infection of schistosome		2	0.0	0.888	Fixed	3.17 (1.92, 5.23)

* *Represents statistical significance (P < 0.05)*.

*OR, odds ratio*.

There were not any statistical significances in these factors such as race, place of birth (rural: OR 1.46, 95% CI: 1.09, 1.96), urinary AFB1 albumin level (OR 2.16, 95% CI: 1.56, 3.00), and infection of schistosome (OR 3.17, 95% CI: 1.92, 5.23).

### Heterogeneity Test

**Figure 2:**
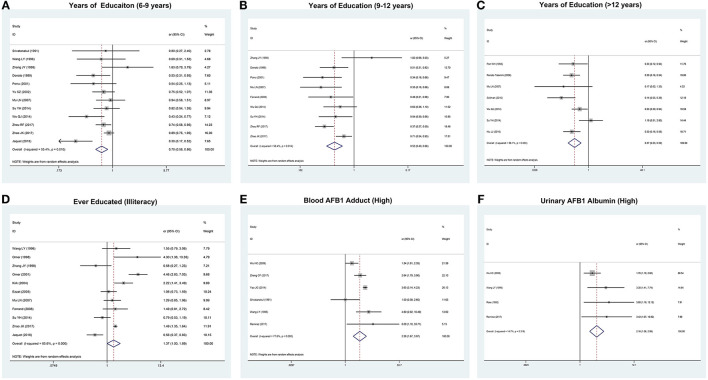
The forest plots of the association between the risk factors and hepatocellular carcinoma (HCC). **(A)** Association between years of education (6–9 years) and HCC. **(B)** Association between years of education (9–12 years) and HCC. **(C)** Association between years of education (>12 years) and HCC. **(D)** Association between ever educated (illiteracy) and HCC. **(E)** Association between blood aflatoxin B1 (AFB1) adduct (high) and HCC. **(F)** Association between urinary AFB1 albumin (high) and HCC.

**Figure 3:**
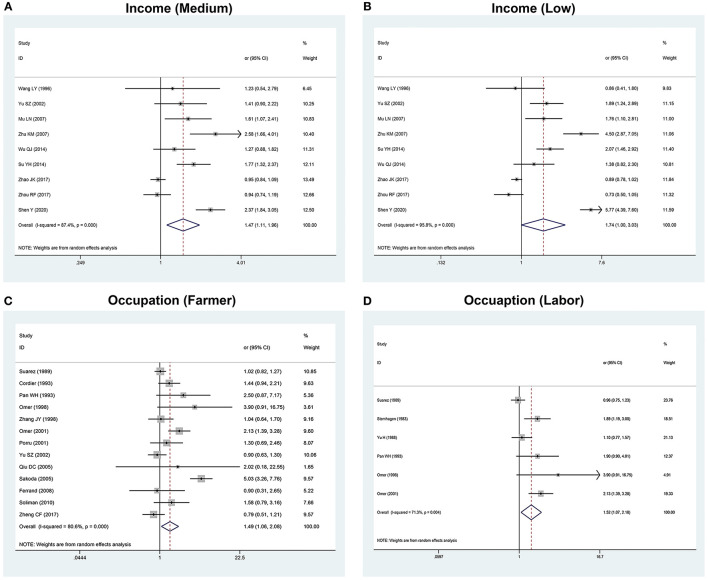
The forest plots of the association between the risk factors and HCC. **(A)** Association between income (medium) and HCC. **(B)** Association between income (low) and HCC. **(C)** Association between occupation (farmer) and HCC. **(D)** Association between occupation (labor) and HCC.

*I*^2^-values of urinary AFB1 albumin level, place of birth, and infection of schistosome are <50%, which could be analyzed by using a fixed-effects model. It suggested that urinary AFB1 albumin level (OR 2.16, 95% CI: 1.56–3.00) and infection of schistosome (OR 3.17, 95% CI: 1.92–5.23) were statistically significant (*P* < 0.05; Table 1). *I*^2^-values of years of education and place of residence were much relatively greater than the former factors. A random-effects model was used for this analysis. *I*^2^-values of ever educated (55.4–85.6%), race (95.2–98.7%), income (87.4–95.8%), occupation (80.6–71.3%), passive smoking (96.8%), marital status (93.5%), blood AFB1 adduct level (77.6%), and exposure of pesticide (81.1%) were much greater than other factors (Table 1). The forest plots are given in Figures 2, 3; Supplementary Figure 1.

### Subgroup Analysis

Stratified meta-analysis was employed to explore the heterogeneity in effect estimates based on the following factors: study design (whether the population in the case group and the control group are paired in this study); source of control population (population of the control group from hospital or community); study quality (evaluation score <8 vs. ≥8 points); and sample size [number of the case group (≥500) vs. the control group (<500)]. The significant factors are shown in Table 2. More details are given in Supplementary Tables 2–5. Most results remained stable and did not reveal any significant changes, except that associations between occupation, income, and HCC across sample size. In relatively large sample size studies, these factors tended to show an insignificant association.

**Table 2:** The result of subgroup analysis of some significant factors.

**Factor**	**No. of studies**	**Heterogeneity test**		**Effect estimate**		
		***I^**2**^* (%)**	***P***	**OR and 95% CI**	***Z***	***P***
**Income (Medium)**						
**Source of control**						
Community	7	80.7	0.000	1.40 (1.05, 1.76)	7.80	0.000
Hospital	2	0.0	0.408	1.63 (1.22, 2.04)	7.77	0.000
**Sample size**						
≥500	7	80.0	0.000	1.62 (1.17, 2.07)	7.08	0.000
<500	2	0.0	0.636	0.96 (0.83, 1.08)	15.06	0.000
**Income (low)**						
**Study design**						
Frequency matched	4	87.4	0.000	3.34 (1.72, 4.95)	4.06	0.000
Individual matched	5	54.0	0.069	0.89 (0.78, 1.00)	16.08	0.000
**Source of control**						
Community	7	88.8	0.000	1.63 (1.07, 2.20)	5.65	0.000
Hospital	2	0.0	0.757	1.99 (1.45, 2.54)	7.17	0.000
**Sample size**						
≥500	7	90.3	0.000	2.31 (1.38, 3.24)	4.86	0.000
<500	2	0.0	0.934	0.89 (0.77, 1.01)	14.39	0.000
**Occupation (farmer)**						
**Study design**						
Frequency matched	9	63.9	0.005	1.29 (0.89, 1.68)	6.35	0.000
Individual matched	4	0.0	0.638	0.99 (0.69, 1.3)	6.45	0.000
**Source of control**						
Community	5	77.5	0.001	2.07 (0.97, 3.17)	3.68	0.000
Hospital	8	0.0	0.667	0.96 (0.77, 1.16)	9.51	0.000
**Quality score**						
≥8	4	62.9	0.000	1.35 (0.85, 1.85)	5.31	0.000
<8	9	0.0	0.517	1.07 (0.87, 1.27)	10.51	0.000
**Sample size**						
≥500	2	91.7	0.001	2.86 (−1.05, 6.78)	1.43	0.152
<500	11	15.8	0.293	1.03 (0.84, 1.22)	10.52	0.000
**Occupation (labor)**						
**Source of control**						
Community	3	67.1	0.048	1.49 (0.42, 2.56)	2.73	0.006
Hospital	3	36.1	0.209	1.27 (0.91, 1.62)	6.94	0.000
**Marital status (married)**						
**Source of control**						
Community	3	0.0	0.888	0.72 (0.59, 0.85)	10.8	0.000
Hospital	3	90.9	0.000	0.51 (0.03, 1.00)	2.09	0.037
**Exposure of pesticide**						
**Source of control**						
Community	2	64.0	0.096	2.27 (0.29, 2.24)	2.55	0.011
Hospital	3	0.0	0.483	1.58 (1.01, 2.15)	5.45	0.000
**Quality score**						
≥8	2	74.6	0.020	1.41 (0.67, 2.15)	3.75	0.000
<8	3	0.0	0.363	1.28 (0.35, 2.22)	2.70	0.007
**Sample size**						
≥500	2	64.0	0.096	0.93 (0.85, 1.01)	24.00	0.000
<500	3	0.0	0.483	1.58 (1.01, 2.15)	5.45	0.000
**Blood AFB1 adduct (high)**						
**Study design**						
Frequency matched	3	91.2	0.000	2.62 (1.20, 4.04)	3.62	0.000
Individual matched	3	32.5	0.227	1.27 (0.17, 2.38)	2.26	0.024
**Quality score**						
≥8	4	87.2	0.000	2.78 (1.44, 4.12)	4.06	0.000
<8	2	0.0	0.542	1.03 (−0.12, 2.17)	1.75	0.080

*OR, odds ratio*.

### Regression Analysis

We incorporated sample size, study design, study quality, and source of control population into regression equation for a comprehensive analysis of the sources of study heterogeneity. Factors that included <10 studies were not tested. The data showed that the source of control population was the heterogeneity source of the factor occupation (farmer). More details are shown in Supplementary Table 6.

### Sensitivity Analysis and Publication Bias

Through sensitivity analysis, we could qualitatively define whether the results of those factors were reliable by comparing the outcomes consistency of a random- and a fixed-effects model. It exhibited that the results of the factors such as education, income, occupation, place of residence, place of birth, blood AFB1 adduct level, urinary AFB1 albumin level, and infection of schistosome are much stable (Supplementary Table 7). Begg's rank correlation test, Egger's linear regression test, and the funnel plot were employed to detect the publication bias. Factors including <10 studies were not tested. Both the results of Begg's and Egger's tests were demonstrated that there was no evidence of publication bias in occupation, ever educated, and 6–9 years of education and the intuitive funnel plot revealed no statistical significance (*P* > 0.05). More details are shown in Supplementary Table 8 and funnel plot are given in Supplementary Figure 2.

## Discussion

This systematic meta-analysis was based on 42 case–control studies with 57,892 participants in 11 countries. Thus, it provides the up-to-date epidemiological evidences that clarifying the association and makes full use of the risk factors related to its incidence rate, reduce the risk of HCC, and provide a scientific basis for prevention and treatment. According to the Global Cancer Statistics 2020, except for the chronic infection with hepatitis B and C virus, the main risk factors for HCC are AF-contaminated foods, heavy alcohol intake, excess body weight, type 2 diabetes, smoking, etc., (1) which are consistent with this study. Abundant clinical and experimental evidences have proved the initiation and progression of HCC, especially environmental and socioeconomic risk factors and have instructed the development of improved, precise modes of prevention and early detection of cancer (16). In fact, this study demonstrates the importance of gene–environment interactions in the multifactorial development of HCC (17). It is now clear that certain occupational, environmental, and lifestyle factors also play a role in cancer development including smoking, alcohol consumption, workplace exposure to vinyl chloride, and exposure to polycyclic aromatic hydrocarbons and AFs (18).

Here, there are two aspects for education. One is that whether the population were educated with a total of 11 studies included. The pooled data showed that compared with the educated population, OR value of the uneducated population was 1.37 (95% CI: 1.00–1.89), which suggested that education was found a protective factor for HCC in this study. The other is involved in the years of education with a total of 17 studies included. When the population with primary education or below as a control group, it demonstrated that, with the increasing of the education time, OR values were gradually decreased, which would reduce the occurrence and development of HCC, suggesting that years of education could also be considered as a protective factor for the incidence of HCC.

In China, the rural population still accounts for a considerable proportion of Chinese population. Due to urban-rural disparity of educational conditions and educational level (19), relatively few people are educated or sufficiently educated, which may promote the occurrence and development of HCC in China. It is reported that education is the protective factor for upper digestive and respiratory sites, stomach, liver, cervix, etc. (20). Popularizing education and allowing more people to receive education cannot only play a role to improve the life quality, but also becomes a potential tool to prevent the prevalence of diseases. Changes in application procedures, packaging, mixing, use of personal protective equipment, and biological monitoring reduced pesticide exposure under controlled conditions (21).

When talking about occupation, it could be divided into two aspects. The first was farmer, with an OR value of 1.49 (95% CI: 1.06–2.08) and *I*^2^-value of 80.6%. The second was labor, with an OR value of 1.52 (95% CI: 1.07–2.18) and *I*^2^-value of 71.3%. All the above results presented statistical significance (*P* < 0.05) and revealed that they were two of the risk factors of HCC. However, the heterogeneities of the two factors in this study were high. Thus, some following analyses were taken in order to figure out the source of heterogeneity. Sensitivity analysis indicated that the results remained coincidence after comparing with the consistency of outcomes of the two effect models, which mean that the results were more reliable. In the stratified analysis and regression analysis, the results showed that the source of the control population was a synergistic heterogeneity source of the occupation factor. Occupation in this study represented the farmer population and labors with hard work and poor economic conditions, which may be the lead cause of HCC. All the participants were divided into three levels by their incomes and we chose the highest level as the control group. OR values of the medium- and low-income groups were 1.48 (95% CI: 1.11–1.96) and 1.74 (95% CI: 1.00–3.03).

The stratified result based on frequency matching of the low-income group was 3.34 (95% CI: 1.72–4.95), showing a high correlation with the occurrence and development of HCC and occupation was highly correlated with income. Most of the farmers and labors belong to the low- and medium-income groups in China and the two results were corresponding to each other. As we know, China, as the largest developing country, is still a large agricultural country. Although China is in the status of rapid development, the disparity of many aspects, especially conditions of economy, medical care, and resources between rural and urban areas, still exists (22). By accelerating the development of rural urbanization to improve the living conditions, incomes, and working environments, the risk of morbidity of populations including farmers and workers at greater social vulnerability is expected and predicted to be reduced.

Pesticides, chemicals employed to manage and treat pests, have been linked to human cancers. Pesticides are widely used or abused in agriculture and horticulture and human exposure primarily occurs via diet. Five studies were incorporated in the analysis of this group. OR value of exposure of pesticide was 1.52 (95% CI: 0.95–2.42) and *I*^2^-value was 81.1%. The sensitivity analysis reported that the result was unstable. After stratified by the source of the control group, the heterogeneity of the result in the hospital control was low, with an OR value of 1.58 (95% CI: 1.01–2.15), indicating that exposure of pesticide is considered as a risk factor for developing HCC, as it is one of the most important reasons for the high incidence rate of HCC in agricultural workers in rural areas. Several points should be reminded in order to prevent liver cancer caused by exposure of pesticide in rural areas. First, government should provide favorable conditions for propaganda, education, and supervision to society for use of highly toxic pesticides, minimize pesticide exposure, and without keeping at home. Second, the use of new-type pesticides with lower toxicity should be encouraged. Finally, it is recommended to follow strict instructions or guidelines for ensuring good health protection against pesticides.

In terms of impact, schistosomiasis is the second only to malaria as the most devastating parasitic disease. People are found to be infected when the skin is in contact with contaminated freshwater by schistosome. Most human infections are caused by *Schistosoma mansoni, Schistosoma haematobium*, or *Schistosoma japonicum* (*S. japonicum*). Only *S. japonicum* is found in China and *Oncomelania* is the only intermediate host of it. As schistosomiasis is a parasitic disease mainly caused by eggs, it enters the portal vein of human liver and gallbladder system. It mainly destroys the intestinal mucosa of the human body, produces secretions, metabolites, and mechanical stimulation, and causes damage to the liver. We have included two studies in our research. Considering it as a risk factor for HCC, OR value was 3.17 (95% CI: 1.92–5.23). The vulnerable groups at most risk are fishermen and farmers. Control measures are to eliminate *Oncomelania* and to strengthen the management of manure and water.

Aflatoxin is a naturally toxic substance with many derivatives (23), among which AFB1 has the highest carcinogenicity (24). Southeast Asia, sub-Saharan Africa, and some parts of South America experience the highest risk of exposure to AFs. AFB1 exposure may be responsible for approximately between 25,200 and 155,000 HCC cases worldwide (25). It is also considered as a major risk factor of HCC in China and many effective measures were taken for removement of the intake in diet, especially in high-endemic areas such as Shanghai City (26). AF compounds in the blood and metabolites in the urine reflect the level of the exposure of AF in the human. In this study, 8 articles were included. The results of AF albumin both in the urine and blood suggested that it was a risk factor for HCC. After exposure to AF, it can be detected in blood, liver, kidney, and urine. But, it is first reflected in the blood, then reaches the organs of the human body, and finally excreted through the urine. Therefore, through detecting the level of AF adduct in blood and urine, we can know the level of internal exposure of AF. AFB1 causes serious harm to human body. Short-term or long-term intakes can cause acute or chronic injury. Warm and humid conditions are the favorable conditions for its growth (27), so the main distribution areas in China are southeast coastal areas, especially in rural regions (28). No matter for people or food, regular monitoring of AF in rural areas is essential. It is of great importance to the early prevention and treatment of liver cancer or other diseases caused by AF.

In terms of place of birth and residence, our emphasis is mainly on rural regions. However, the number of the included studies were two and three, respectively. Here, we make a concise analysis. OR value of birth place in rural region was 1.46 (1.09, 1.96) and the *I*^2^-value was 0.0%. The result was stable, suggesting that it was significantly associated with the occurrence and development of HCC. Residence place in rural region had an OR value of 1.05 (0.76, 1.44) and the *I*^2^-value of 61.3%. Although the degree of heterogeneity was relatively high, the sensitivity analysis showed that it was reliable. Therefore, it also suggested that there was no significant correlation between the occurrence and development of HCC. However, there are still many rural areas all over the world including China. Many factors such as food storage methods, eating habit, drinking water, exposure of the pesticide, and working for a long time may also lead to the occurrence of HCC. From the above results, we found being a farmer or a labor was a risk factor for HCC (*P* < 0.05). As far as we know, studies included for synthesis might be too limited to reflect the real situation. More samples and further studies are still required in the future.

In these factors, analysis for racial divisions were included as White (as the control), Black (OR: 2.42, 95% CI: 1.10, 5.31), Hispanic (OR: 1.90, 95% CI: 0.87, 4.17), and Asian (OR: 5.36, 95% CI: 0.72, 40.14), the marital status (OR: 0.69, 95% CI: 0.28, 1.69), and the passive smoking (OR: 1.43, 95% CI: 0.27, 7.51). For these abovementioned factors, the sensitivity analysis results remained unstable. All the races by stratified analysis indicate no statistical significance (*P* > 0.05). Hence, there are no obvious correlations between these three factors and the occurrence and development of HCC.

## Conclusion

Generally, our meta-analysis reveals that the environmental and socioeconomic risk factors including ever educated (illiteracy); race (Black, Hispanic, and Asian); medium and low incomes; occupations (farmer and labor); passive smoking; place of residence (rural); blood AFB1 adduct level; exposure of pesticide, etc. were statistically increased with the occurrence and development of HCC. Moreover, our findings suggested that it is critically pivotal to improve effective policies and programs for national education level, the increasing of public and physician awareness, the acceleration of urbanization process in the high-risk populations and areas especially in rural areas, and elimination and reduction of the differences between urban and rural areas including medical and educational resources and levels, healthcare services, economic income, employment, etc. All of these are effective means for prevention and control of HCC.

## Data Availability Statement

The datasets presented in this study can be found in online repositories. The names of the repository/repositories and accession number(s) can be found in the article/Supplementary Material.

## Author Contributions

FZ, DT, and NL were involved in the administrative support, conception and design of the manuscript, data interpretation, reviewed, and revised the manuscript. WL was involved in the conception and design of the manuscript, collection and assembly of the data, data analysis, and drafted the initial manuscript. ZL, RZ, and LD were involved in the collection and assembly of data and drafted the initial manuscript. WX, WC, and RZ were involved in the conception and design of the manuscript, reviewed, and revised the manuscript. YC and YT were involved in the conception, design of the manuscript, and data interpretation. ZL and LL were involved in critically reviewed the manuscript for important intellectual content. All the authors contributed to the article and approved the submitted version of the manuscript.

## Funding

The authors gratefully acknowledge the financial support by the National Natural Science Foundation of China (No. 81872584), the National 863 Young Scientist Program (No. 2015AA020940), the Natural Science Foundation of Guangdong Province (No. 2016A030313138), Key Projects of Guangzhou Science and Technology Program (No. 201704020056), the Natural Science Foundation of Shenzhen (No. JCYJ20210324093211030), the Medical Scientific Research Foundation of Guangdong Province (No. A2020490), Military Logistics Research Project (No. CKJ20J031), and Interdisciplinary Research for First-class Discipline Construction Project of Henan University (No. 2019YLXKJC04).

## Conflict of Interest

The authors declare that the research was conducted in the absence of any commercial or financial relationships that could be construed as a potential conflict of interest.

## Publisher's Note

All claims expressed in this article are solely those of the authors and do not necessarily represent those of their affiliated organizations, or those of the publisher, the editors and the reviewers. Any product that may be evaluated in this article, or claim that may be made by its manufacturer, is not guaranteed or endorsed by the publisher.
