# Supplementary material for: Association Between Environmental and Socioeconomic Risk Factors and Hepatocellular Carcinoma: A Meta-Analysis
Source: Front Public Health. 2022 Feb 18;10:741490. doi: 10.3389/fpubh.2022.741490 (PMC8893961; doi:10.3389/fpubh.2022.741490)
Supplement: Supplementary file 1 [file Data_Sheet_1.docx]

ONLINE **SUPPLEMENTAL MATERIAL**

**Association Between Environmental and Socioeconomic Risk Factors and Hepatocellular Carcinoma: A Meta-Analysis**

Wenfeng Lu^1, 4^, Fengjiao Zheng^2,^ *
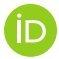
, Zhi Li^1, 4^, Rui Zhou^1, 4^, Lugang Deng^1, 4^, Wenwei Xiao^1, 4^, Wenyan Chen^1, 4^, Rong Zhao^1, 4^, Yulan Chen^1, 4^, Yuxing Tan^1, 4^, Zhibo Li^1, 4^, Limin Liu^3, 4^, Duxun Tan ^1^ *, Nan Liu^1, 3, 4^ *
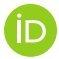


^1^ *Institute of Environment and Health, South China Hospital, Health Science Center, Shenzhen University, Shenzhen, 518116, P. R. China.*

^2^ *Department of Clinical Laboratory, The Air Force Hospital of Southern Theater Command of PLA, Guangzhou, 510602, P. R. China.*

^3^ *Institute of Chronic Disease Risks Assessment, School of Nursing and Health, Henan University, Kaifeng, 475004, P. R. China.*

^4^ *College of Public Health, Zhengzhou University, Zhengzhou, 540001, P. R. China.*

PROSPERO Registration: Association Between Environmental Factors and Hepatocellular Carcinoma: A Meta-Analysis, crd.york.ac.uk/prospero/, ID: CRD42020151710

**Correspondence**

*Fengjiao Zheng*, Department of Clinical Laboratory, The Air Force Hospital of Southern Theater Command of PLA, 510602, P. R. China

Email: [fjzheng458@126.com](mailto:fjzheng458@126.com)

*Duxun Tan*, Institute of Environment and Health, South China Hospital, Health Science Center, Shenzhen University, Shenzhen, 518116, P. R. China.

Email: [2968456616@qq.com](mailto:2968456616@qq.com)

*Nan Liu*, Institute of Environment and Health, South China Hospital, Health Science Center, Shenzhen University, Shenzhen, 518116, P. R. China.

Email: [13688869875@163.com](mailto:13688869875@163.com)

**Contents:**

**Table S1** The basic characteristics and the quality evaluation of the included studies.

**Table S2** The result of subgroup analysis stratified by study design.

**Table S3** The result of subgroup analysis stratified by source of control.

**Table S4** The result of subgroup analysis stratified by quality of studies.

**Table S5** The result of subgroup analysis stratified by sample size.

**Table S6** The result of meta regression.

**Table S7** The result of sensitivity test.

**Table S8** Publication bias measured by Begg’s and Egger’s test.

**Figure S1** Funnel plot for factors of race-Black (A), race-Hispanic (B), race-Asian (C), place of residence (D), place of birth (E), exposure of pesticide (F), marital status (G), passive smoking (H), infection of schistosome (I).

**Figure S2** Funnel plot for factors of years of education (A), ever educated (B) and occupation-farmer (C).

**Table S1** The basic characteristics of the included studies

| **No.** | **First Author** | **Publication Year** | **Research Year** | **Country** | **Sex** | **Study Design** | **Source of Control** | **Case** | **Control** | **Matching Factors** | **Selection** | **Comparability** | **Exposure** | **Total** |
| --- | --- | --- | --- | --- | --- | --- | --- | --- | --- | --- | --- | --- | --- | --- |
| 1 | Stemhagen [1] | 1983 | 1975-1980 | USA | M/F | Individual matched | Hospital | 265 | 530 | Occupation | 3 | 2 | 2 | 7 |
| 2 | Kew [2] | 1986 | - | Africa | M/F | Individual matched | - | 392 | 392 | - | 1 | 2 | 0 | 3 |
| 3 | Yu [3] | 1988 | 1969-1985 | USA | M/F | Individual matched | Hospital | 165 | 465 | Occupation, Race | 2 | 2 | 2 | 6 |
| 4 | Suarez [4] | 1989 | 1969-1980 | USA | M/F | Frequency matched | Community | 1742 | 1742 | Occupation | 3 | 2 | 2 | 7 |
| 5 | Srivatanakul [5] | 1991 | 1987-1988 | Thailand | M/F | Individual matched | Community | 65 | 65 | Education Blood AFB1 adduct, | 3 | 2 | 2 | 7 |
| 6 | Ross [6] | 1992 | 1986-1990 | China | M/F | Frequency matched | Community | 22 | 140 | Urinary AFB1 albumin | 4 | 2 | 3 | 9 |
| 7 | Pan [7] | 1993 | 1988-1989 | China | M/F | Frequency matched | Hospital | 59 | 101 | Education, Occupation | 2 | 2 | 2 | 6 |
| 8 | Cordier [8] | 1993 | 1989-1992 | Vietnam | M | Frequency matched | Hospital | 152 | 241 | Occupation, Exposure of pesticide | 3 | 1 | 2 | 6 |
| 9 | Wang [9] | 1996 | 1991-1995 | China | M/F | Individual matched | Community | 56 | 220 | Education, Income, Blood AFB1 adduct, Urinary AFB1 albumin | 4 | 2 | 3 | 9 |
| 10 | Braga [10] | 1997 | 1984-1993 | Italy | M/F | Frequency matched | Hospital | 320 | 1408 | - | 3 | 0 | 2 | 5 |
| 11 | Omer [11] | 1998 | 1995 | Sudan | M/F | Frequency matched | Community | 24 | 34 | Education, Occupation | 4 | 2 | 3 | 9 |
| 12 | Zhang [12] | 1998 | 1994-1995 | China | M/F | Frequency matched | Hospital | 152 | 115 | Education, Occupation, Exposure of pesticide | 3 | 2 | 2 | 7 |
| 13 | Donato [13] | 1999 | 1996-1998 | Italy | M/F | Individual matched | Hospital | 284 | 464 | Education | 3 | 2 | 3 | 8 |
| 14 | Omer [14] | 2001 | 1996-1998 | Sudan | M/F | Frequency matched | Community | 150 | 205 | Education, Occupation | 4 | 0 | 3 | 7 |
| 15 | Porru [15] | 2001 | 1997-1999 | Italy | M | Individual matched | Hospital | 144 | 283 | Education, Occupation | 3 | 2 | 3 | 8 |
| 16 | Yu SZ [16] | 2002 | 1995-1997 | China | M/F | Individual matched | Hospital | 248 | 248 | Education, Occupation, Income | 4 | 2 | 3 | 9 |
| 17 | Kirk [17] | 2004 | 1997-2001 | Gambia | M/F | Frequency matched | Hospital | 216 | 408 | Education | 3 | 2 | 3 | 8 |
| 18 | Ezzat [18] | 2005 | - | Egypt | M/F | Individual matched | Hospital | 236 | 236 | Education, Place of birth, Exposure of pesticide, Marital status | 3 | 2 | 3 | 8 |
| 19 | Sakoda [19] | 2005 | 1992-2000 | China | M/F | Frequency matched | Community | 166 | 394 | Occupation | 3 | 2 | 3 | 8 |
| 20 | Qiu [20] | 2005 | 1995-2002 | China | M/F | Individual matched | Hospital | 127 | 127 | Occupation, Infection of schistosome | 4 | 2 | 3 | 9 |
| 21 | Talamini [21] | 2006 | 1999-2002 | Italy | M/F | Frequency matched | Hospital | 185 | 412 | Education | 3 | 2 | 3 | 8 |
| 22 | Mu [22] | 2007 | 2000 | China | M/F | Frequency matched | Community | 204 | 415 | Education, Income | 4 | 2 | 3 | 9 |
| 23 | Zhu [23] | 2007 | 1984-1988 | USA | M/F | Frequency matched | Community | 168 | 1910 | Income, Race, Marital status | 3 | 2 | 2 | 7 |
| 24 | Ferrand [24] | 2008 | 2000-2003 | French | M | Frequency matched | Hospital | 125 | 142 | Education, Occupation | 3 | 2 | 3 | 8 |
| 25 | Hassan [25] | 2008 | 2000-2006 | USA | M/F | Frequency matched | Hospital | 319 | 1061 | Race, Passive smoking, Marital status | 3 | 2 | 3 | 8 |
| 26 | Wu [26] | 2009 | 1991-2004 | China | M/F | Frequency matched | Community | 230 | 1052 | Blood AFB1 adduct, Urinary AFB1 albumin | 3 | 2 | 3 | 8 |
| 27 | Soliman [27] | 2010 | 2007-2009 | Egypt | M/F | Individual matched | Community | 150 | 150 | Education, Occupation, Place of residence, Passive smoking, Marital status, Infection of schistosome | 4 | 2 | 3 | 9 |
| 28 | Zhao [28] | 2012 | 2007-2009 | China | M/F | Frequency matched | Hospital | 345 | 961 | - | 2 | 0 | 3 | 5 |
| 29 | Lai [29] | 2014 | 1994-2013 | China | M/F | Frequency matched | Community | 68 | 150 | - | 2 | 2 | 3 | 7 |
| 30 | Wu [30] | 2014 | 2002-2008 | China | M/F | Individual matched | Community | 217 | 427 | Education, Income | 4 | 2 | 3 | 9 |
| 31 | Yao [31] | 2014 | 2004-2012 | China | M/F | Frequency matched | Hospital | 1486 | 1996 | Blood AFB1 adduct | 4 | 2 | 3 | 9 |
| 32 | Su [32] | 2014 | 2007-2009 | China | M/F | Frequency matched | Hospital | 345 | 961 | Education, Income, Marital status | 2 | 0 | 3 | 5 |
| 33 | Vopham [33] | 2015 | 2000-2009 | USA | M/F | Frequency matched | Community | 3034 | 14991 | Race, Place of residence, Exposure of pesticide | 4 | 2 | 3 | 9 |
| 34 | Niu [34] | 2016 | 2011-2014 | China | M/F | Individual matched | Community | 314 | 346 | Education, Exposure of pesticide, Passive smoking | 3 | 2 | 3 | 8 |
| 35 | Zhao [35] | 2017 | 2003-2010 | China | M/F | Individual matched | Community | 2018 | 8019 | Education, Income | 3 | 2 | 2 | 7 |
| 36 | Ramirez [36] | 2017 | 2012-2014 | USA | M/F | Individual matched | Community | 51 | 104 | Blood AFB1 adduct, Urinary AFB1 albumin | 4 | 2 | 3 | 9 |
| 37 | Zheng [37] | 2017 | 2013-2016 | China | M/F | Frequency matched | Hospital | 214 | 214 | Occupation, Blood AFB1 adduct | 3 | 2 | 3 | 8 |
| 38 | Zhou [38] | 2017 | 2013-2016 | China | M/F | Individual matched | Community | 644 | 644 | Education, Income | 4 | 2 | 3 | 9 |
| 39 | Jaquet [39] | 2018 | - | Africa | M/F | Individual matched | Hospital | 160 | 320 | Education | 3 | 2 | 3 | 8 |
| 40 | Mak [40] | 2018 | 2000-2012 | Africa | M/F | Individual matched | Hospital | 150 | 438 | Place of residence, Place of birth | 2 | 2 | 3 | 7 |
| 41 | Chu [41] | 2018 | 1991-2011 | China | M/F | Individual Matched | Community | 203 | 1943 | Blood AFB1 adduct level | 4 | 2 | 3 | 9 |
| 42 | Shen [42] | 2020 | 2011-2016 | USA | M/F | Frequency matched | Community | 673 | 1166 | Race, Marital status, Income | 3 | 2 | 3 | 8 |

Factors including education, occupation, income, marital status, race, place of residence, place of birth, exposure of pesticide, passive smoking, infection of schistosome, blood AFB1 adduct, and urinary AFB1 albumin.

**References**

1. Stemhagen A, Slade J, Altman R, Bill J. Occupational risk factors and liver cancer. A retrospective case-control study of primary liver cancer in New Jersey. Am J Epidemiol. 1983;117(4):443-54.

2. Kew MC, Kassianides C, Hodkinson J, Coppin A, Paterson AC. Hepatocellular carcinoma in urban born blacks: frequency and relation to hepatitis B virus infection. BMJ. 1986;293(6558):1339-41.

3. Yu H, Harris RE, Kabat GC, Wynder EL, %J International Journal of Cancer Journal International Du Cancer. Cigarette smoking, alcohol consumption and primary liver cancer: a case-control study in the USA. Int J Cancer. 1988;42(3):325-8.

4. Suarez L, Weiss NS, Martin J. Primary liver cancer death and occupation in Texas. Am J Ind Med. 1989;15(2):167-75.

5. Srivatanakul P, Parkin DM, Khlat M, Chenvidhya D, Chotiwan P, Insiripong S, L'Abbe KA, Wild CP. Liver cancer in Thailand. II. A case-control study of hepatocellular carcinoma. Int J Cancer. 1991;48(3):329-32.

6. Ross RK, Yuan JM, Yu MC, Wogan GN, Qian GS, Tu JT, Groopman JD, Gao YT, Henderson BE. Urinary aflatoxin biomarkers and risk of hepatocellular carcinoma. Lancet. 1992;339(8799):943-6.

7. Pan WH, Wang CY, Huang SM, Yeh SY, Lin WG, Lin DI, Liaw YF. Vitamin A, Vitamin E or beta-carotene status and hepatitis B-related hepatocellular carcinoma. Ann Epidemiol. 1993;3(3):217-24.

8. Cordier S, Le TB, Verger P, Bard D, Le CD, Larouze B, Dazza MC, Hoang TQ, Abenhaim L. Viral infections and chemical exposures as risk factors for hepatocellular carcinoma in Vietnam. Int J Cancer. 1993;55(2):196-201.

9. Wang LY, Hatch M, Chen CJ, Levin B, You SL, Lu SN, Wu MH, Wu WP, Wang LW, Wang Q *et al*. Aflatoxin exposure and risk of hepatocellular carcinoma in Taiwan. Int J Cancer. 1996;67(5):620-5.

10. Braga C, La Vecchia C, Negri E, Franceschi S. Attributable risks for hepatocellular carcinoma in northern Italy. Eur J Cancer. 1997;33(4):629-34.

11. Omer RE, Bakker MI, van't Veer P, Hoogenboom RL, Polman TH, Alink GM, Idris MO, Kadaru AM, Kok FJ. Aflatoxin and liver cancer in Sudan. Nutr Cancer. 1998;32(3):174-80.

12. Zhang JY, Wang X, Han SG, Zhuang H. A case-control study of risk factors for hepatocellular carcinoma in Henan, China. Am J Trop Med Hyg. 1998;59(6):947-51.

13. Donato F, Gelatti U, Chiesa R, Albertini A, Bucella E, Boffetta P, Tagger A, Ribero ML, Portera G, Fasola M *et al*. A case-control study on family history of liver cancer as a risk factor for hepatocellular carcinoma in North Italy. Brescia HCC Study. Cancer Causes Control. 1999;10(5):417-21.

14. Omer RE, Verhoef L, Van't Veer P, Idris MO, Kadaru AM, Kampman E, Bunschoten A, Kok FJ. Peanut butter intake, GSTM1 genotype and hepatocellular carcinoma: a case-control study in Sudan. Cancer Causes Control. 2001;12(1):23-32.

15. Porru S, Placidi D, Carta A, Gelatti U, Ribero ML, Tagger A, Boffetta P, Donato F. Primary liver cancer and occupation in men: a case-control study in a high-incidence area in Northern Italy. Int J Cancer. 2001;94(6):878-83.

16. Yu SZ, Huang XE, Koide T, Cheng G, Chen GC, Harada K, Ueno Y, Sueoka E, Oda H, Tashiro F *et al*. Hepatitis B and C viruses infection, lifestyle and genetic polymorphisms as risk factors for hepatocellular carcinoma in Haimen, China. Jpn J Cancer Res. 2002;93(12):1287-92.

17. Kirk GD, Lesi OA, Mendy M, Akano AO, Sam O, Goedert JJ, Hainaut P, Hall AJ, Whittle H, Montesano R. The Gambia Liver Cancer Study: Infection with hepatitis B and C and the risk of hepatocellular carcinoma in West Africa. Hepatology. 2004;39(1):211-9.

18. Ezzat S, Abdel-Hamid M, Eissa SA, Mokhtar N, Labib NA, El-Ghorory L, Mikhail NN, Abdel-Hamid A, Hifnawy T, Strickland GT *et al*. Associations of pesticides, HCV, HBV, and hepatocellular carcinoma in Egypt. Int J Hyg Environ Health. 2005;208(5):329-39.

19. Sakoda LC, Graubard BI, Evans AA, London WT, Lin WY, Shen FM, McGlynn KA. Toenail selenium and risk of hepatocellular carcinoma mortality in Haimen City, China. Int J Cancer. 2005;115(4):618-24.

20. Qiu DC, Hubbard AE, Zhong B, Zhang Y, Spear RC. A matched, case-control study of the association between Schistosoma japonicum and liver and colon cancers, in rural China. Ann Trop Med Parasitol. 2005;99(1):47-52.

21. Talamini R, Polesel J, Montella M, Dal Maso L, Crispo A, Tommasi LG, Izzo F, Crovatto M, La Vecchia C, Franceschi S. Food groups and risk of hepatocellular carcinoma: A multicenter case-control study in Italy. Int J Cancer. 2006;119(12):2916-21.

22. Mu LN, Cao W, Zhang ZF, Cai L, Jiang QW, You NC, Goldstein BY, Wei GR, Chen CW, Lu QY *et al*. Methylenetetrahydrofolate reductase (MTHFR) C677T and A1298C polymorphisms and the risk of primary hepatocellular carcinoma (HCC) in a Chinese population. Cancer Causes Control. 2007;18(6):665-75.

23. Zhu K, Moriarty C, Caplan LS, Levine RS. Cigarette smoking and primary liver cancer: a population-based case-control study in US men. Cancer Causes Control. 2007;18(3):315-21.

24. Ferrand JF, Cenee S, Laurent-Puig P, Loriot MA, Trinchet JC, Degos F, Bronovicky JP, Pelletier G, Stucker I. Hepatocellular carcinoma and occupation in men: a case-control study. J Occup Environ Med. 2008;50(2):212-20.

25. Hassan MM, Spitz MR, Thomas MB, El-Deeb AS, Glover KY, Nguyen NT, Chan W, Kaseb A, Curley SA, Vauthey JN *et al*. Effect of different types of smoking and synergism with hepatitis C virus on risk of hepatocellular carcinoma in American men and women: case-control study. Int J Cancer. 2008;123(8):1883-91.

26. Wu HC, Wang Q, Yang HI, Ahsan H, Tsai WY, Wang LY, Chen SY, Chen CJ, Santella RM. Aflatoxin B1 exposure, hepatitis B virus infection, and hepatocellular carcinoma in Taiwan. Cancer Epidemiol Biomarkers Prev. 2009;18(3):846-53.

27. Soliman AS, Hung CW, Tsodikov A, Seifeldin IA, Ramadan M, Al-Gamal D, Schiefelbein EL, Thummalapally P, Dey S, Ismail K. Epidemiologic risk factors of hepatocellular carcinoma in a rural region of Egypt. Hepatol Int. 2010;4(4):681-90.

28. Zhao B, Shen H, Liu F, Liu S, Niu J, Guo F, Sun X. Exposure to organochlorine pesticides is an independent risk factor of hepatocellular carcinoma: a case-control study. J Expo Sci Environ Epidemiol. 2012;22(6):541-8.

29. Lai H, Mo X, Yang Y, He K, Xiao J, Liu C, Chen J, Lin Y. Association between aflatoxin B1 occupational airway exposure and risk of hepatocellular carcinoma: a case-control study. Tumour Biol. 2014;35(10):9577-84.

30. Wu QJ, Wang J, Gao J, Zhang W, Han LH, Gao S, Gao YT, Ji BT, Zheng W, Shu XO *et al*. Urinary isothiocyanates level and liver cancer risk: a nested case-control study in Shanghai, China. Nutr Cancer. 2014;66(6):1023-9.

31. Yao JG, Huang XY, Long XD. Interaction of DNA repair gene polymorphisms and aflatoxin B1 in the risk of hepatocellular carcinoma. Int J Clin Exp Pathol. 2014;7(9):6231-44.

32. Su Y, Zhao B, Guo F, Bin Z, Yang Y, Liu S, Han Y, Niu J, Ke X, Wang N *et al*. Interaction of benzo[a]pyrene with other risk factors in hepatocellular carcinoma: a case-control study in Xiamen, China. Ann Epidemiol. 2014;24(2):98-103.

33. Vopham T, Brooks MM, Yuan JM, Talbott EO, Ruddell D, Hart JE, Chang CC, Weissfeld JL. Pesticide exposure and hepatocellular carcinoma risk: A case-control study using a geographic information system (GIS) to link SEER-Medicare and California pesticide data. Environ Res. 2015;143(Pt A):68-82.

34. Niu J, Lin Y, Guo Z, Niu M, Su C. The Epidemiological Investigation on the Risk Factors of Hepatocellular Carcinoma: A Case-Control Study in Southeast China. Medicine. 2016;95(6):e2758.

35. Zhao JK, Wu M, Kim CH, Jin ZY, Zhou JY, Han RQ, Yang J, Zhang XF, Wang XS, Liu AM *et al*. Jiangsu Four Cancers Study: a large case-control study of lung, liver, stomach, and esophageal cancers in Jiangsu Province, China. Eur J Cancer Prev. 2017;26(4):357-64.

36. Ramirez AG, Munoz E, Parma DL, Michalek JE, Holden AEC, Phillips TD, Pollock BH. Lifestyle and Clinical Correlates of Hepatocellular Carcinoma in South Texas: A Matched Case-control Study. Clin Gastroenterol Hepatol. 2017;15(8):1311-2.

37. Zheng C, Zeng H, Lin H, Wang J, Feng X, Qiu Z, Chen JA, Luo J, Luo Y, Huang Y *et al*. Serum microcystin levels positively linked with risk of hepatocellular carcinoma: A case-control study in southwest China. Hepatology. 2017;66(5):1519-28.

38. Zhou RF, Chen XL, Zhou ZG, Zhang YJ, Lan QY, Liao GC, Chen YM, Zhu HL. Higher dietary intakes of choline and betaine are associated with a lower risk of primary liver cancer: a case-control study. Sci Rep. 2017;7(1):679.

39. Jaquet A, Tchounga B, Tanon A, Bagny A, Ekouevi DK, Traore HA, Sasco AJ, Maiga M, Dabis F. Etiology of hepatocellular carcinoma in West Africa, a case-control study. Int J Cancer. 2018;143(4):869-77.

40. Mak D, Babb de Villiers C, Chasela C, Urban MI, Kramvis A. Analysis of risk factors associated with hepatocellular carcinoma in black South Africans: 2000-2012. PLoS One. 2018;13(5):e0196057.

41. Chu YJ, Yang HI, Wu HC, Lee MH, Liu J, Wang LY, Lu SN, Jen CL, You SL, Santella RM *et al*. Aflatoxin B1 exposure increases the risk of hepatocellular carcinoma associated with hepatitis C virus infection or alcohol consumption. Eur J Cancer. 2018;9437-46.

42. Shen Y, Risch H, Lu L, Ma X, Irwin ML, Lim JK, Taddei T, Pawlish K, Stroup A, Brown R *et al*. Risk factors for hepatocellular carcinoma (HCC) in the northeast of the United States: results of a case-control study. Cancer Causes Control. 2020.

**Table S2** The result of subgroup analysis stratified by study design

| Factor | Study Design | No of studies | Heterogeneity Test | | Effect Estimate | | |
| --- | --- | --- | --- | --- | --- | --- | --- |
|  |  |  | I^2^ (%) | *P* | OR and 95%CI | Z | *P* |
| Ever educated (Illiteracy) | Frequency matched | 7 | 73.5 | 0.001 | 1.38 (0.81, 1.95) | 4.73 | 0.000 |
|  | Individual matched | 4 | 93.4 | 0.000 | 1.12 (0.52, 1.72) | 3.65 | 0.000 |
| Income (Medium) | Frequency matched | 4 | 31.0 | 0.226 | 1.97 (1.64, 2.30) | 11.75 | 0.000 |
|  | Individual matched | 5 | 0.0 | 0.477 | 0.98 (0.88, 1.09) | 18.32 | 0.000 |
| Income (Low) * | Frequency matched | 4 | 87.4 | 0.000 | 3.34 (1.72, 4.95) | 4.06 | 0.000 |
|  | Individual matched | 5 | 54.0 | 0.069 | 0.89 (0.78, 1.00) | 16.08 | 0.000 |
| Occupation (Farmer) * | Frequency matched | 9 | 63.9 | 0.005 | 1.29 (0.89, 1.68) | 6.35 | 0.000 |
|  | Individual matched | 4 | 0.0 | 0.638 | 0.99 (0.69, 1.3) | 6.45 | 0.000 |
| Occupation (Labor) | Frequency matched | 4 | 58.7 | 0.064 | 1.05 (0.82, 1.28) | 8.97 | 0.000 |
|  | Individual matched | 2 | 59.2 | 0.117 | 1.23 (0.86, 1.6) | 6.56 | 0.000 |
| Marital status (Married) | Frequency matched | 4 | 95.0 | 0.000 | 0.52 (0.17, 0.87) | 1.63 | 0.104 |
|  | Individual matched | 2 | 78.5 | 0.031 | 1.75 (-0.36, 3.86) | 2.93 | 0.003 |
| Exposure of pesticide | Frequency matched | 3 | 0.0 | 0.499 | 0.93 (0.85, 1.00) | 24.00 | 0.000 |
|  | Individual matched | 2 | 0.0 | 0.755 | 1.82 (1.19, 2.44) | 5.72 | 0.000 |
| Blood AFB1 albumin (High) * | Frequency matched | 3 | 91.2 | 0.000 | 2.62 (1.20, 4.04) | 3.62 | 0.000 |
|  | Individual matched | 3 | 32.5 | 0.227 | 1.27 (0.17, 2.38) | 2.26 | 0.024 |

** Represents statistical significance.*

*OR, odds ratio.*

**Table S3** The result of subgroup analysis stratified by source of control

| Factor | Source of Control | No. of studies | Heterogeneity Test | | Effect Estimate | | |
| --- | --- | --- | --- | --- | --- | --- | --- |
|  |  |  | I^2^ (%) | *P* | OR and 95%CI | Z | *P* |
| Ever educated (Illiteracy) | Community | 5 | 53.3 | 0.073 | 1.49 (1.35, 1.63) | 5.96 | 0.000 |
|  | Hospital | 6 | 67.7 | 0.009 | 0.92 (0.59, 2.24) | 5.46 | 0.000 |
| Race (Black) | Community | 3 | 91.3 | 0.000 | 3.21 (0.49, 5.94) | 2.31 | 0.021 |
|  | Hospital | 2 | 80.2 | 0.025 | 2.16 (0.05, 4.26) | 2.01 | 0.044 |
| Income (Medium) * | Community | 7 | 80.7 | 0.000 | 1.40 (1.05, 1.76) | 7.80 | 0.000 |
|  | Hospital | 2 | 0.0 | 0.408 | 1.63 (1.22, 2.04) | 7.77 | 0.000 |
| Income (Low) * | Community | 7 | 88.8 | 0.000 | 1.63 (1.07, 2.20) | 5.65 | 0.000 |
|  | Hospital | 2 | 0.0 | 0.757 | 1.99 (1.45, 2.54) | 7.17 | 0.000 |
| Occupation (Farmer) * | Community | 5 | 77.5 | 0.001 | 2.07 (0.97, 3.17) | 3.68 | 0.000 |
|  | Hospital | 8 | 0.0 | 0.667 | 0.96 (0.77, 1.16) | 9.51 | 0.000 |
| Occupation (Labor) * | Community | 3 | 67.1 | 0.048 | 1.49 (0.42, 2.56) | 2.73 | 0.006 |
|  | Hospital | 3 | 36.1 | 0.209 | 1.27 (0.91, 1.62) | 6.94 | 0.000 |
| Marital status (Married) * | Community | 3 | 0.0 | 0.888 | 0.72 (0.59, 0.85) | 10.8 | 0.000 |
|  | Hospital | 3 | 90.9 | 0.000 | 0.51 (0.03, 1.00) | 2.09 | 0.037 |
| Exposure of pesticide * | Community | 2 | 64.0 | 0.096 | 2.27 (0.29, 2.24) | 2.55 | 0.011 |
|  | Hospital | 3 | 0.0 | 0.483 | 1.58 (1.01, 2.15) | 5.45 | 0.000 |
| Blood AFB1 albumin (High) | Community | 4 | 4.0 | 0.373 | 1.47 (0.89, 2.04) | 4.99 | 0.000 |
|  | Hospital | 2 | 63.5 | 0.098 | 3.44 (2.96, 3.92) | 13.94 | 0.000 |

** Represents statistical significance.*

*OR, odds ratio.*

**Table S4** The result of subgroup analysis stratified by quality of studies.

| Factor | Quality of Studies | No. of Studies | Heterogeneity Test | | Effect Estimate | | |
| --- | --- | --- | --- | --- | --- | --- | --- |
|  |  |  | I^2^ (%) | *P* | OR and 95%CI | Z | *P* |
| Ever educated (Illiteracy) | ≥8 | 9 | 74.7 | 0.000 | 1.26 (0.84, 1.68) | 5.92 | 0.000 |
|  | <8 | 2 | 92 | 0.000 | 1.06 (0.18, 1.95) | 2.35 | 0.019 |
| Race (Black) | ≥8 | 3 | 91.1 | 0.000 | 3.23 (0.45, 6.01) | 2.28 | 0.023 |
|  | <8 | 2 | 81.3 | 0.021 | 2.16 (0.08, 4.23) | 2.04 | 0.042 |
| Income (Medium) | ≥8 | 6 | 80.2 | 0.000 | 1.55 (1.04, 2.06) | 6.01 | 0.000 |
|  | <8 | 3 | 76.8 | 0.013 | 1.33 (0.75, 1.90) | 4.53 | 0.000 |
| Income (Low) | ≥8 | 6 | 89.0 | 0.000 | 1.83 (0.97, 2.69) | 4.17 | 0.000 |
|  | <8 | 3 | 90.5 | 0.000 | 2.10 (0.72, 3.48) | 2.97 | 0.003 |
| Occupation (Farmer) * | ≥8 | 4 | 62.9 | 0.000 | 1.35 (0.85, 1.85) | 5.31 | 0.000 |
|  | <8 | 9 | 0.0 | 0.517 | 1.07 (0.87, 1.27) | 10.51 | 0.000 |
| Occupation (Labor) | ≥8 | 2 | 0.0 | 0.662 | 2.16 (1.22, 3.10) | 4.51 | 0.000 |
|  | <8 | 4 | 40.7 | 0.167 | 1.06 (0.87, 1.25) | 10.37 | 0.000 |
| Exposure of pesticide * | ≥8 | 2 | 74.6 | 0.020 | 1.41 (0.67, 2.15) | 3.75 | 0.000 |
|  | <8 | 3 | 0.0 | 0.363 | 1.28 (0.35, 2.22) | 2.70 | 0.007 |
| Blood AFB1 adduct (High) * | ≥8 | 4 | 87.2 | 0.000 | 2.78 (1.44, 4.12) | 4.06 | 0.000 |
|  | <8 | 2 | 0.0 | 0.542 | 1.03 (-0.12, 2.17) | 1.75 | 0.080 |

* Represents statistical significance (*P*<0.05).

*OR, odds ratio.*

**Table S5** The result of subgroup analysis stratified by sample size.

| Factor | N | No. of Studies | Heterogeneity Test | | Effect Estimate | | | |
| --- | --- | --- | --- | --- | --- | --- | --- | --- |
|  |  |  | I^2^ (%) | *P* | OR and 95%CI | Z | *P* | |
| Ever educated (Illiteracy) | ≥500 | 4 | 82.1 | 0.001 | 1.12 (0.62, 1.62) | 4.38 | 0.000 |  |
|  | <500 | 7 | 73.0 | 0.001 | 1.33 (0.86, 1.80) | 5.56 | 0.000 |  |
| Income (Medium) * | ≥500 | 7 | 80.0 | 0.000 | 1.62 (1.17, 2.07) | 7.08 | 0.000 |  |
|  | <500 | 2 | 0.0 | 0.636 | 0.96 (0.83, 1.08) | 15.06 | 0.000 |  |
| Income (Low) * | ≥500 | 7 | 90.3 | 0.000 | 2.31 (1.38, 3.24) | 4.86 | 0.000 |  |
|  | <500 | 2 | 0.0 | 0.934 | 0.89 (0.77, 1.01) | 14.39 | 0.000 |  |
| Occupation (Farmer) * | ≥500 | 2 | 91.7 | 0.001 | 2.86 (-1.05, 6.78) | 1.43 | 0.152 |  |
|  | <500 | 11 | 15.8 | 0.293 | 1.03 (0.84, 1.22) | 10.52 | 0.000 |  |
| Occupation (Labor) | ≥500 | 3 | 48.7 | 0.142 | 1.04 (0.84, 1.24) | 10.15 | 0.000 |  |
|  | <500 | 3 | 0.0 | 0.873 | 2.09 (1.29, 2.89) | 5.10 | 0.000 |  |
| Marital status (Married) | ≥500 | 4 | 95.0 | 0.000 | 0.52 (0.17, 0.87) | 2.93 | 0.003 | |
|  | <500 | 2 | 78.5 | 0.031 | 1.75 (-0.36, 3.86) | 1.63 | 0.104 | |
| Exposure of pesticide * | ≥500 | 2 | 64.0 | 0.096 | 0.93 (0.85, 1.01) | 24.00 | 0.000 | |
|  | <500 | 3 | 0.0 | 0.483 | 1.58 (1.01, 2.15) | 5.45 | 0.000 | |
| Blood AFB1 adduct (High) | ≥500 | 2 | 95.5 | 0.000 | 2.60 (0.54, 4.67) | 2.47 | 0.013 | |
|  | <500 | 4 | 50.3 | 0.110 | 1.99 (1.22, 2.75) | 5.10 | 0.000 | |

** Represents statistical significance (P<0.05).*

*OR, odds ratio.*

**Table S6** The result of meta regression.

| Factor | Analysis Factor | t | *P* |
| --- | --- | --- | --- |
| Year of education (6-9) | N | 0.60 | 0.564 |
|  | Score | -0.92 | 0.387 |
|  | Study design | -1.54 | 0.168 |
|  | Source of control | -0.10 | 0.926 |
| Ever educated (Illiteracy) | N | 0.56 | 0.593 |
|  | Score | 0.93 | 0.386 |
|  | Study design | -1.16 | 0.289 |
|  | Source of control | -1.83 | 0.116 |
| Occupation (Farmer) | N | -2.16 | 0.063 |
|  | Score | -0.52 | 0.614 |
|  | Study design | -0.25 | 0.809 |
|  | Source of control | -2.51 | 0.036 * |

** Represents statistical significance (P<0.05).*

*N, sample size.*

**Table S7** The result of sensitivity test.

| Factor | | Random-effect Model OR (95%CI) | Fixed- effect Model OR (95% CI) |
| --- | --- | --- | --- |
| Years of education | 0,0-6 | 1 | 1 |
|  | 6-9 | 0.70 (0.58, 0.86) | 0.77 (0.69, 0.86) * |
|  | 9-12 | 0.52 (0.40, 0.68) | 0.53 (0.45, 0.61) * |
|  | ＞12 | 0.37 (0.23, 0.59) | 0.38 (0.28, 0.51) * |
| Ever educated (Illiteracy) | | 1.37 (1.00, 1.89) | 1.42 (1.31, 1.55) * |
| Race | White | 1 | 1 |
|  | Black | 2.42 (1.10, 5.31) | 1.61 (1.40, 1.87) |
|  | Hispanic | 1.90 (0.87, 4.17) | 1.21 (1.06, 1.38) |
|  | Asian | 5.36 (0.72, 40.14) | 1.16 (1.06, 1.27) |
| Income | High | 1 | 1 |
|  | Medium | 1.48 (1.11, 1.96) | 1.24 (1.14, 1.36) * |
|  | Low | 1.74 (1.00, 3.03) | 1.42 (1.28, 1.56) * |
| Occupation (Farmer) | | 1.49 (1.06, 2.08) | 1.31 (1.15, 1.49) * |
| Occupation (Labor) | | 1.52 (1.07, 2.18) | 1.28 (1.08, 1.51) * |
| Passive smoking | | 1.43 (0.27, 7.51) | 2.15 (1.66, 2.80) |
| Marital status (Married) | | 0.68 (0.36, 1.29) | 0.62 (0.53, 0.71) |
| Place of residence (Rural) | | 1.05 (0.76, 1.44) | 0.98 (0.85, 1.13) * |
| Place of birth (Rural) | | 0.86 (0.73, 1.02) | 0.86 (0.73, 1.02) * |
| Blood AFB1 adduct (High)^1^ | | 2.58 (1.67, 3.97) | 3.21 (2.82, 3.65) * |
| Urinary AFB1 albumin (High)^1^ | | 2.32 (1.56, 3.45) | 2.16 (1.56, 3.00) * |
| Exposure of pesticide | | 1.52 (0.95, 2.42) | 0.97 (0.90, 1.05) |
| Infection of schistosome | | 3.17 (1.92, 5.23) | 3.17 (1.92, 5.23) * |

** Represents statistical significance. 1, low or undetectable. OR, odds ratio. AFB1 aflatoxin B1.*

**Table S8** Publication bias measured by Begg’s and Egger’s test.

| Factor | Begg’ s Test | | Egger’s Test | |
| --- | --- | --- | --- | --- |
|  | Z | *P* | t | *P* |
| Year of education (6-9) | 0.07 | 0.945 | -1.37 | 0.199 |
| Ever educated (Illiteracy) | 0.93 | 0.350 | -0.26 | 0.803 |
| Occupation (Farmer) | 1.40 | 0.161 | 1.06 | 0.310 |

**Supplementary Figures**


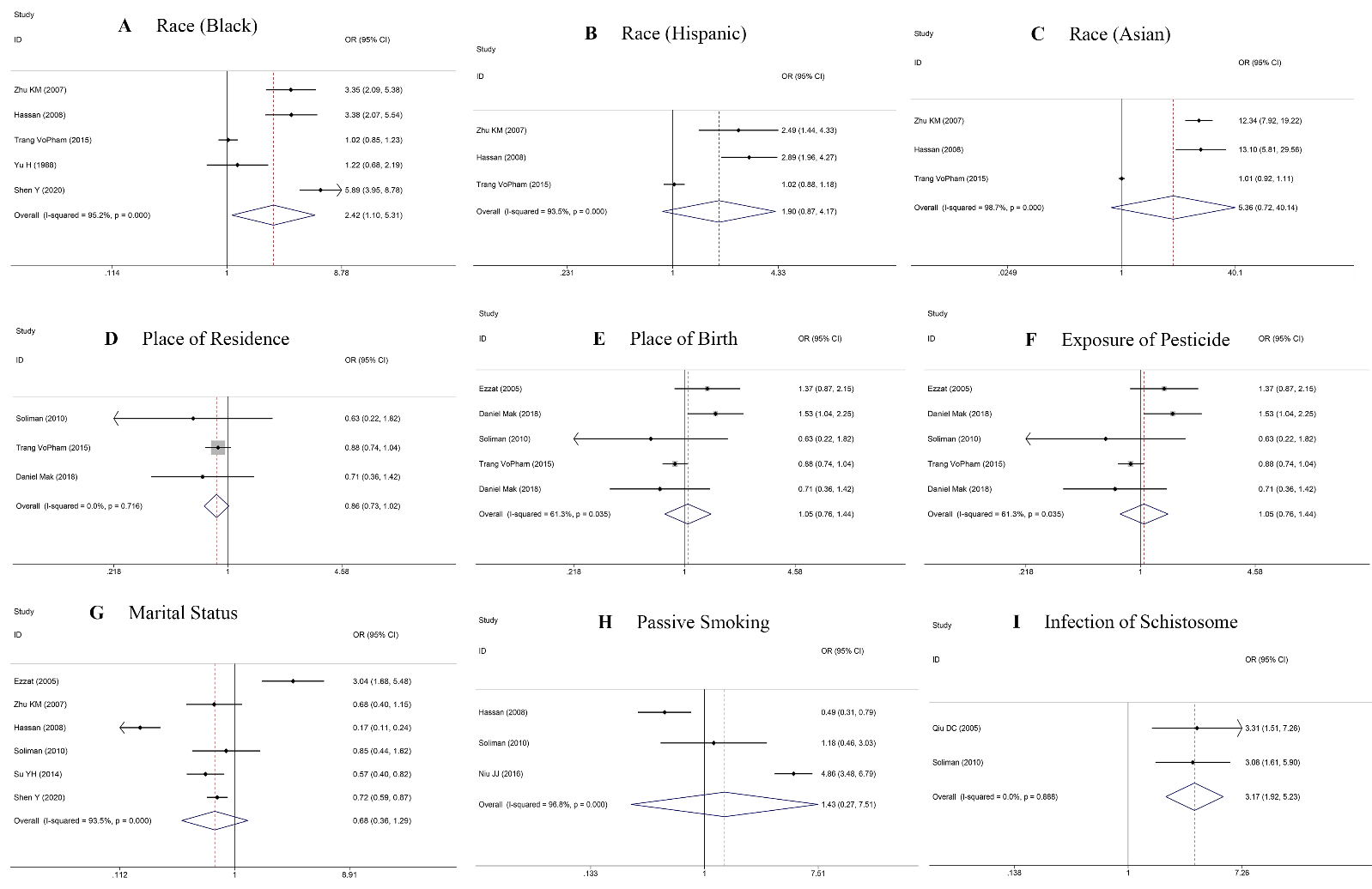


**Figure S1** Funnel plot for factors of race-Black (A), race-Hispanic (B), race-Asian (C), place of residence (D), place of birth (E), exposure of pesticide (F), marital status (G), passive smoking (H), infection of schistosome (I).


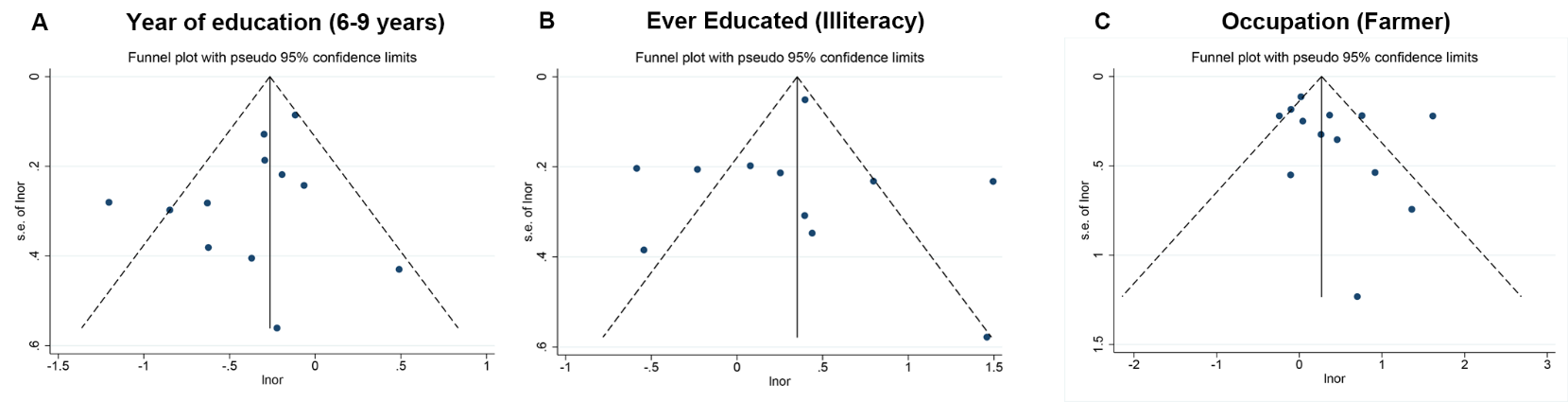


**Figure S2** Funnel plot for factors of years of education (A), ever educated (B) and occupation-farmer (C).
